# Ascites—VI

**Published:** 1909-07-17

**Authors:** 


### Medicine.

### ASCITES—VI.

### THE DIFFERENTIAL DIAGNOSIS OF THE CAUSE OF ASCITES (*Continued*).

(c) *Tuberculous Peritonitis.*—This disease is the commonest cause of ascites in children. There are several varieties, which may be classified according to the morbid changes found in the abdomen. Ascites may be found with each of these forms:—

(i) The peritoneum, both parietal and visceral, may be studded with tubercles which are often most numerous in the flanks and on the under surface of the diaphragm. The abdomen becomes uniformly distended, partly as a result of ascites, and partly from tympanites. The symptoms of this form of the complaint may be acute or chronic.

In the acute form there is abdominal distention, pain, and tenderness; the temperature may rise to 104° F.; the pulse becomes rapid, and the condition may be mistaken for acute suppurative peritonitis. The onset, however, is seldom so acute as in acute peritonitis due to appendicitis or to perforated gastric ulcer, and the abdominal pain and tenderness are less marked. When the onset is not quite so acute, and the illness begins with a general feeling of malaise, abdominal discomfort, distension, pain, and tenderness, with pyrexia, and a pulse-rate above normal, it may be difficult to distinguish it from typhoid fever. In both diseases there may be leucopenia, and the spleen may bo moderately enlarged.

The pulse-rate will probably be a useful clinical guide, for this is not so rapid as would be proportional to the temperature in typhoid fever. For instance, with a temperature of 102° F., the pulse-rate would very likely be only 90 in the earlier stages of typhoid fever, whereas, with the same temperature, it might well be 110 or more in acute tuberculous peritonitis. If the presence of free fluid in the peritoneal cavity can be determined it would be very important evidence in favour of tuberculous peritonitis and against typhoid fever. In the more chronic forms, in which the abdomen gradually increases in size and the chief indication of disease is the presence of ascites, it may be either very easy or very difficult to make a correct diagnosis. The condition is constantly being mistaken for cirrhosis of the liver on the one hand and for ovarian tumour with secondary peritonitis upon the other. The frequent association of tuberculosis with chronic alcoholism increases the difficulties of diagnosis in adults.

(ii) The omentum may be contracted and thickened as a result of tuberculous infiltration and caseation, so that it gives rise to a hard tumour lying transversely across the abdomen above the umbilicus in such a way as to simulate an enlarged liver. From the latter it may often be distinguished by the presence of a resonant percussion note between its upper limit and the costal margin. In some cases the edge of the liver may be felt above it and distinct from it. This variety may also be mistaken for malignant peritonitis; but as a matter of fact a thickened omentum is commoner in tuberculosis than in cancer. It may, however, be mistaken for a liver studded with large umbilicated secondary nodules.

(iii) The intestines may be matted together, and the adhesions thickened and infiltrated with tuberculous deposits. In consequence, the peritoneal cavity may be divided into loculi, some containing fluid. In encysted ascites of this kind the distension is often asymmetrical.

(iv) The mesentery may be thickened and contracted, the intestines being fixed to the posterior part of the peritoneal cavity. The abnormal and atypical physical signs that may arise in consequence of this have already been discussed. The abdomen may be uniformly distended and dull to percussion; occasionally a large, hard, irregular, deeply situated tumour may be felt, perhaps by dipping.

(v) The mesenteric glands may be enlarged and caseous, with irregular shaped tumours palpable in the posterior part of the abdomen. Abdominal pains, chronic pyrexia, and wasting in a young subject may be the only evidence. Ascites is by no means a constant, nor even a common, feature of this form of tuberculous peritonitis.

(vi) Occasionally local thickening of the abdominal wall may be felt, due to subperitoneal inflammatory and tuberculous infiltrations. This causes a hard rigid feeling in the abdominal parietes, like firm local contractions of the recti.

There may also be redness and œdema for some little distance around the umbilicus, and even a purulent or fæco-purulent discharge from it. When present these are very characteristic of tuberculous peritonitis. Wasting and diarrhœa are apt to occur, and there may be co-existent pleurisy.

(d) *Cancerous Peritonitis.*—Cancerous peritonitis occurs most frequently in women over 40 years of age. It is usually secondary to cancer of the stomach, ovary, intestine, or other abdominal viscus. The omentum may be thickened and infiltrated with the new growth, and large nodules may develop all over the peritoneum. The chief indications of the presence of this disease are ascites, emaciation, anæmia, and cachexia. If the amount of fluid is large, nothing definite in the form of nodules or tumours can be made out on palpation, on account of the tenseness of the abdominal wall. After paracentesis abdominis it may be possible to feel nodules and masses varying in size and form. Roughly speaking, such multiple nodules in the abdomen in a patient past middle life indicate malignant disease, whilst similar nodules in a child point to tubercle. In many instances the ascitic fluid may contain an appreciable amount of blood. This is always suggestive, though not quite pathognomonic, of growth. It is when no primary growth is known of that the difficulties occur. The state of the left supraclavicular glands may sometimes give the clue. The umbilicus may be infiltrated with growth, becoming hard, fixed, and much thickened. This condition is almost pathognomonic.

